# Neural Constrained Charge Equilibration for Extracting Charge Transport Parameters from Machine Learned Interatomic Potentials

**DOI:** 10.1021/acsomega.6c04842

**Published:** 2026-10-13

**Authors:** Carson Farmer, Hector Medina

**Affiliations:** 5199 School of Engineering Liberty University Lynchburg, Virginia 24515, United States

## Abstract

Machine-learned interatomic potentials (MLIPs) continue to gain popularity as efficient tools for predicting energies and forces for atomic structures at an accuracy comparable to quantum chemistry. However, there are limited trained MLIPs for certain important applications such as in charge transport. Accelerating and automating the determination of parameters for such applications can be of critical importance in the rapid discovery of materials such as organic electronics (e.g., OLEDs). This work develops and introduces neural constrained charge equilibriation (NcQEq) and applies the technique to AimNet2 and OrbMol-V2. The new method enables extracting information about the diabatic states and thereby charge transport parameters. The approach is demonstrated on model systems against constrained density functional theory charge transport and then tested on intramolecular hole transport properties for polyethylene. This work demonstrates that the NcQeq approach is compatible with the charge equilibration schemes for AimNet2 and the latent charge predictions of OrbMol-V2. After calibration to reference data, the method enables estimation of the electronic coupling, reorganization energy, and the free energy difference.

## Graphical abstract

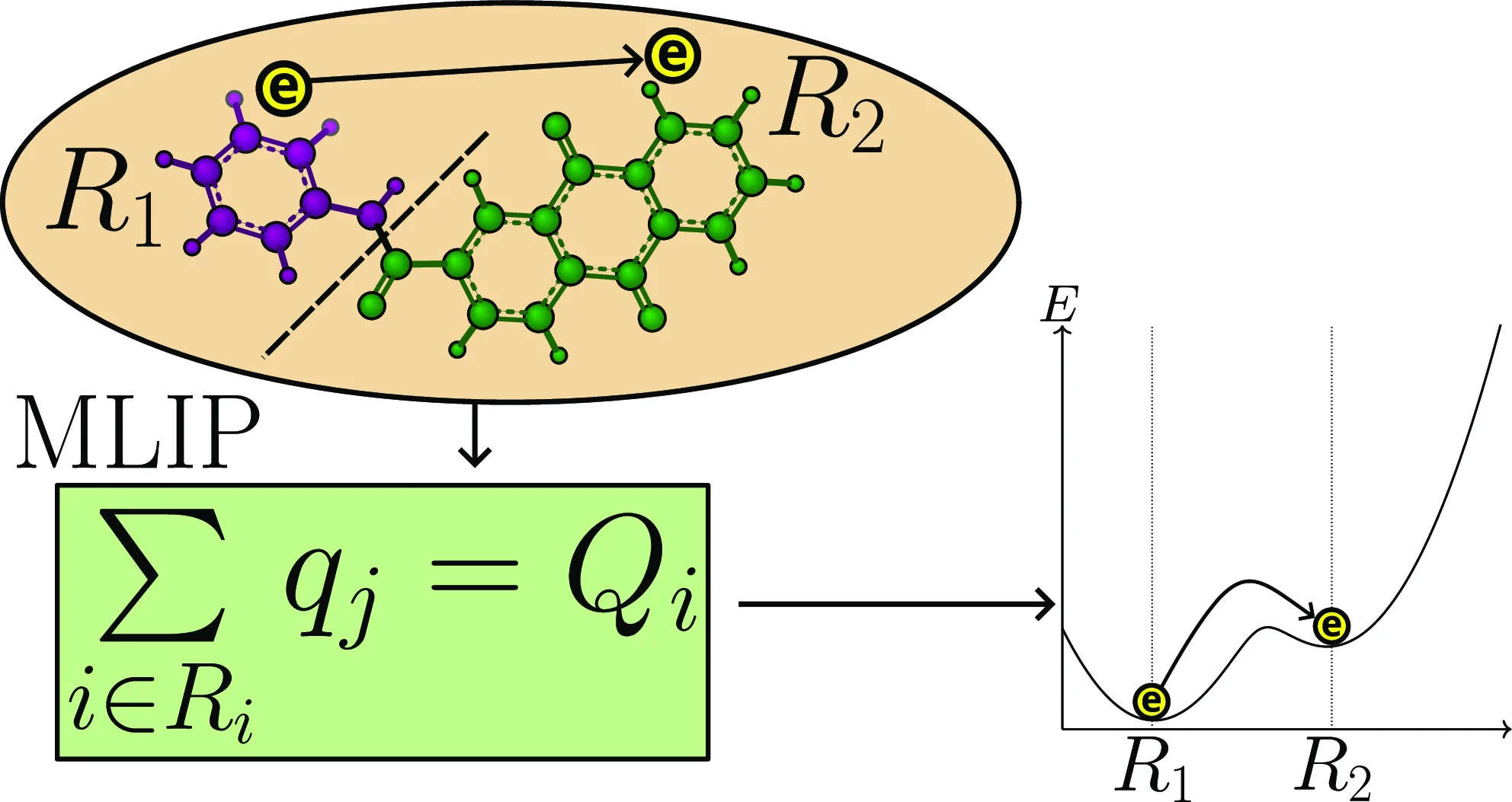

## 1 Introduction

Molecular modeling of charge transfer (CT) enables developing new optoelectronic,^1^ semiconducting,^2^ and dielectric materials.^3–6^ Often, these applications are governed by Marcus theory for adiabatic charge transport.^7^ The requirements for modeling the Marcus transport include estimating the reorganization energy, λ, free energy difference, Δ*G*, and electronic coupling, *V*_12_, for the transition from the reactant to product states. When applying Marcus theory to charge transport, this expression involves the redistribution of charges in a molecular system, where the initial and final states are either the charge separated states or CT states. The current methods require considerable time for estimating the three parameters from computational chemistry calculations.

Commonly λ and Δ*G* are determined using Nelson’s four point method.^8^ Furthermore, additional methods have been developed for estimating *V*_12_ between the diabatic CT states such as the site energy difference,^9^ generalized Mulliken-Hush,^10^ and fragment orbital density functional theory (DFT).^11^ For the four-point method, four separate calculations are required with two of the calculations being geometry optimization operations. For *V*_12_ estimation methods at least a single calculation and oftentimes multiple computational chemistry methods at high-levels of theory are required.^10,12,13^

Recently, Kundu, Ye, and Berkelbach^14^ devised a method based on charge equilibration (QEq) which is capable of predicting the diabatic states using only the electrostatic contributions. With a similarity to the constrained DFT (cDFT) approaches developed by Wu and Van Voorhis,^15,16^ the application of the constraints to the electronic structure problem enables a direct extraction of the Marcus parameters from electronic structure calculations. With next-generation of machine learned interatomic potential (MLIP)­s including partial charge predictions, the modeling of charged-dependent properties such the polarization response^17^ and small molecule collision cross sections,^18^ to name a few applications, has become computationally feasible. The improvements have stemmed from applying either charge-equilibrium like approaches,^19,20^ self-consistent implementations,^21^ or other approaches.^22,23^ The improvements to the modeling framework allows for modeling the long-range interactions where the charge state is coupled with the geometry. The inclusion of partial charge improves long-range behavior and improves the prediction of structures where CT is important.^24^

This work builds on the neural charge equilibration (NQE) in AimNet2 ^20^ and the latent charge predictions in OrbMol-V2 to extract CT properties through a process termed neural constrained charge equilibration (NcQEq). By applying regional constraints to the charge update layers of each model, the CT parameters for Marcus Theory can be approximated. Within charge-transport workflows, this process is demonstrated to replace costly DFT calculations (see Figure 1). The systems tested include modeling charge separation in the formanilide-anthraquinone (FA-AQ) molecule, charge transport in the organic complex - anthracene-tetracyanoethylene (TCNE) (A-TCNE), and charge transport for intrachain hole transport in polyethylene (PE). All calculations are benchmarked against cDFT and are found to estimate the parameters after calibration.

**Figure 1:**
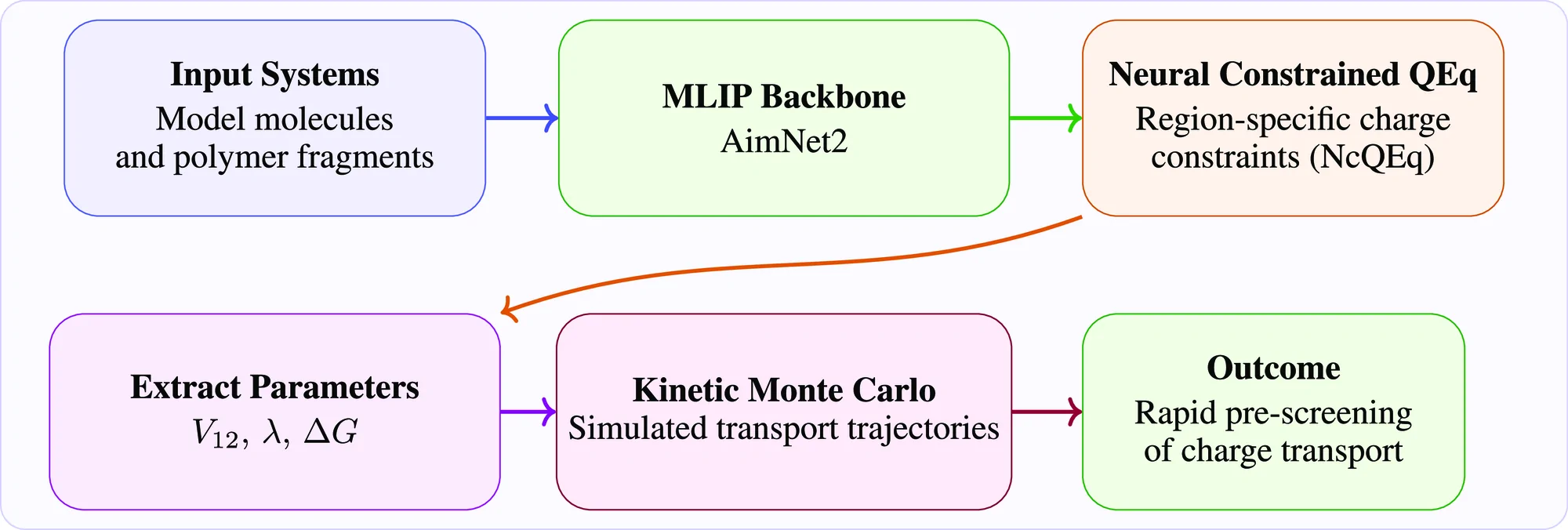
Starting from the geometry input and fragment definitions, the ground state energy is calculated and then charge constraints are applied to create the diabatic CT states in the system. The parameters for charge transport are determined and may be used in downstream tasks such as kinetic Monte Carlo which enables charge transport screening with rapid parametrization.

## 2 Methods

In charge-constrained methods, such as cDFT and constrained charge equilibration (cQEq), a regional constraint on the sum of the partial charges for a group of atoms is applied. In the case of cDFT, the Kohn–Sham equations are modified to include an external biasing potential to apply the constraint. In cQEq, the net charge constraint is applied to the region during the variational minimization part of the Qeq procedure. Both can be viewed as applying a regional constraint to the net charge on a group of atoms. NcQeq applies a similar principle by applying a regional constraint on the sum of the partial charges predicted by a MLIP without performing the engergy minimization portion of Qeq Since different models implement the partial charge prediction and updates differently, this work focuses on the In both AimNet2 and OrbMol-V2, the net charge for the system is constrained internally for each layer. In AimNet2, to achieve a system with a net charge, the excess charge predicted is distributed by a weighting factor across the system to achieve a system with the desired net charge. In OrbMol-V2, the excess charge is distributed evenly.

### 2.1 Constrained Charge Equilibration in AimNet2

In the spirit of cDFT, the charge update function in AimNet2 is modified to have the charge per defined region equal to given charge constraint, *Q*_Region_. Specifically, the AimNet2–2025 model is adapted for this study as it had additional intermolecular data added to the training set which is expected to improve performance for intermolecular interactions.^25^ Specifically, the model checkpoint AimNet2-2025_0 is utilized. To predict the partial charge, *q*_*i*_ for atom *i*, the NQE equation is adjust to

1 $$q_{i}={\mathrm{q̃}}_{i}+\frac{f_{i}}{\sum_{j\in Region}f_{j}}\left(Q_{Region}-\sum_{j\in Region}{\mathrm{q̃}}_{j}\right)$$

where region denotes the selected set of atoms where the constraint is applied, ${\mathrm{q̃}}_{i}$ is the unconstrained and unequilibrated atomic partial charge for atom, *i*, and *f*_*i*_ is the atom-dependent redistribution weight for atom, *i*, and is determined as part of the update procedure in the AimNet2 model. The normalization term, $\sum_{j\in Region}f_{j}$ , ensures that the redistributed charge correction is partitioned across atoms in the selected region.

### 2.2 Constrained Charge Equilibration in OrbMol-V2

The next model study with regional charge constraints is the OrbMol-V2 model which is based on the Orb-v3 architecture^26^ with an additional latent charge prediction branch. The weights for the model are from the checkpoint orbmol-v2-teqabfhg-20260523. The model has an update equation for the charge branch of

2 $$q_{i}={\mathrm{q̃}}_{i}-\frac{1}{N_{atom}}\sum_{j}{\mathrm{q̃}}_{j}+\frac{Q_{net}}{N_{atom}}$$

where *N*_atom_ is the number of atoms in the system and *Q*_net_ is the net charge of the system. To enforce a region charge constraint, the equation is modified to

3 $$q_{i}={\mathrm{q̃}}_{i}-\frac{1}{N_{region}}\sum_{j\in Region}{\mathrm{q̃}}_{j}+\frac{Q_{Region}}{N_{region}}$$

where *N*_region_ is the number of atoms in each region. Rather than applying a weighted distribution of the excess charge in system, OrbMol-V2 evenly distributes the excess charge across the system to reach the correct net charge or regional charge.

#### 2.2.1 Electronic Coupling

The electronic coupling, *V*_12_ is computed from constrained-charge diabatic states following the cQeq formulation of Kundu et al.^14^ and a cDFT-style coupling extraction protocol. For each nuclear geometry, we evaluate reactant, *DA*_*r*_, and product, *DA*_*p*_, constrained CT states along with the adiabatic energy, *U*_0_, evaluated at the unconstrained geometry, *Q*, to estimate $\left|V_{12}\right|$

4 $$\left|V_{12}\right|=\sqrt{\left[U_{0}\left(Q\right)-E\left(DA_{r}|Q\right)\right]\left[U_{0}\left(Q\right)-E\left(DA_{p}|Q\right)\right]}$$

where *DA*_*r*_ is the constraint for the reactant state and *DA*_*p*_ is the constraint for the product state. In this notation, *E*(*x*|*y*) represents the energy for the constrained charge state *x* evaluated at geometry *y*, where *y* denotes a nuclear geometry (for example, *Q*) rather than an electronic-state label. As noted in^15^ this equation may overestimate the *V*_12_ if the adiabatic state is a mixture of the diabats. For the *V*_12_ calculations, the geometries are single-point evaluations for the structure represented as *Q*. This approximation for *V*_12_ may result in a systematic overestimation due to self-interaction errors. While including some quantum chemical insight such as the orbital overlap may improve the accuracy, herein, the focus is accurately reproducing the results expected from the cDFT approach and future works will build on improving the estimate using additional information.

#### 2.2.2 Reorganization Energy and Free Energy Difference

For determining λ and Δ*G*, the approximations from Wu and Van Voorhis^27^ are used

5 $$\lambda =E\left(DA_{p}|DA_{r}\right)-E\left(DA_{p}|DA_{p}\right)$$

and

6 $$\Delta G=E\left(DA_{r}|DA_{r}\right)-E\left(DA_{p}|DA_{p}\right)$$

where the geometries for *DA*_*r*_ and *DA*_*p*_ are the optimized geometries for the respective applied charge constraints.

#### 2.2.3 Data Generation

To benchmark the MLIPs against DFT data, the ωB97M-V functional^28^ with the def2-TZVPD basis set^29^ is selected. AimNet2 was trained on data ωB97M-D3/def-TZVPP and then further trained with B97–3c data. OrbMol-V2 was trained on the OMol-25 and OPoly-26 data sets which are at the ωB97M-V/def2-TZVPD level of theory. Therefore, we expect that the selection of ωB97M-V/def2-TZVPD is sufficient to be used in comparing the NcQeq approach to the cDFT results. The populations are determined using Löwdin analysis. The cDFT is implemented in GPU4PySCF^30^ with a (75,590) grid with SG1 for the VV10 functional. When performing geometry optimizations using cDFT in this work, NWChem is used with ωB97x-D3/def2-TZVP.

For the PE system, an amorphous structure of 20 chains with 50 CH_2_ units per chain is generated. The GAFF force field^31^ and 21-step equilibration process from.^32^ The carrier fragments are determined from the backbone dihedrals using the approach described by Unge et al.^33^

## 3 Results and Discussion

### 3.1 Formanilide-Anthraquinone

Based on the case presented in Wu and Van Voorhis,^27^ the FA-AQ (see Figure 2a) system represents an inverted region for CT whereby the reaction rate decreases with increasing Δ*G*. Based on the results of Okamoto et al.,^34^ FA-AQ exhibits a charge separated state and a prolonged charge separation. To predict the Δ*G* and λ, the molecule is partitioned as shown in Figure 2b. For the *DA*_*r*_ state, both the donor and acceptor are constrained to a net charge of 0. For the *DA*_*p*_ state, the donor is constrained to a + 1 net charge while the acceptor is constrained to a −1 net charge. Using ([Disp-formula eq6]) and ([Disp-formula eq5]), the Δ*G* and λ are predicted and provided in Table 1. For Δ*G*, AimNet2 closely approximates the B3LYP results from,^27^ OrbMol-V2 is more accurate when compared to the reference ωB97M-V and experimental data. Overall, OrbMol-V2 performs similarly to the ωB97M-V cDFT reference data in comparison to the AimNet2 model.

**Figure 2:**
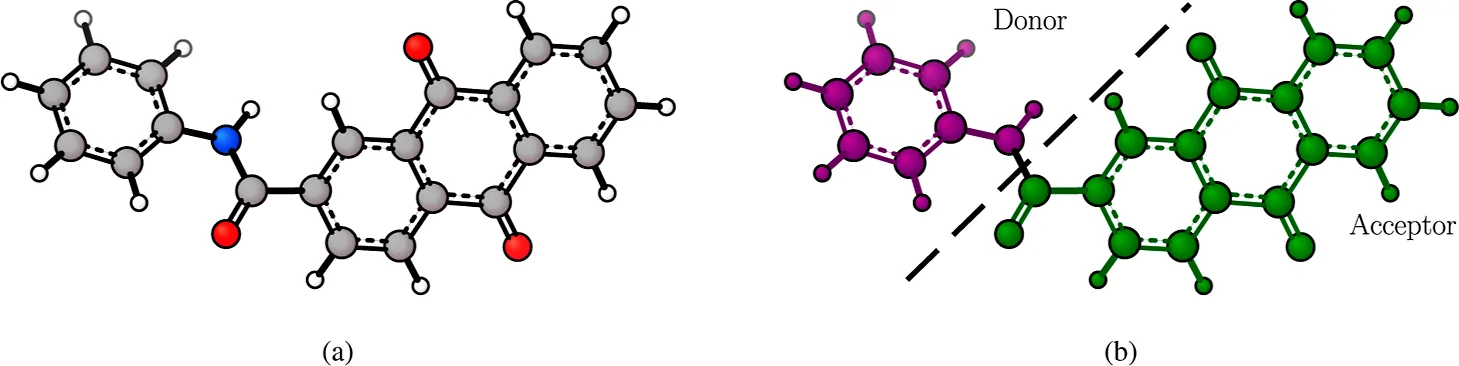
(a) The structure of the FA-AQ molecule used and the partitioning scheme (b) where anthraquinone group serves as the electron acceptor. *DA*_*r*_ is modeled having the electron localized to the left side (purple) and the *DA*_*p*_ involves the electron localized to the right side (green) of the molecule.

**Table 1:** Comparison of the Predicted and Computational Results From^27^ for the Charge Separated State of FA-AQ^a^

method	–Δ*G* [eV]	λ [eV]
experimental^34^	2.24	-
cDFT (B3LYP/6-31 + G(d))^27^	3.25	0.26
cDFT (ωB97M-V/def2-TZVPD)	2.17	0.22
NcQeq—AimNet2	3.63	0.27
NcQeq—OrbMol-V2	2.21	0.15

a The results are reported as the negative of Δ*G* to align with those in^27^ an additional calculation was performed at the ωB97M-V/def2-TZVPD level of theory.

### 3.2 Anthracene-Tetracyanoethylene

Next, using an example system from Kundu et al.,^14^ the A-TCNE complex is studied. The system is partitioned such that the *DA*_*r*_ is the complex where both the anthracene and *DA*_*p*_ is defined as the anthracene molecule having a net charge of +1 and the TCNE molecule having a net charge of −1. The structure is shown in Figure 3. The adiabatic energies and constrained state energies are shown in Figure 4 and Figure 5.

**Figure 3:**
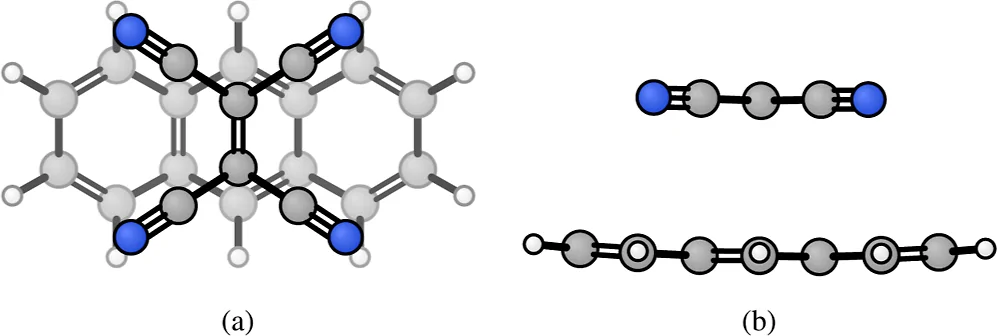
Anthracene-tetracyanoethylene complex views used for analysis where CT occurs from the anthracene to the TCNE through the vacuum region. The top view is shown in (a) and a side view in (b).

**Figure 4:**
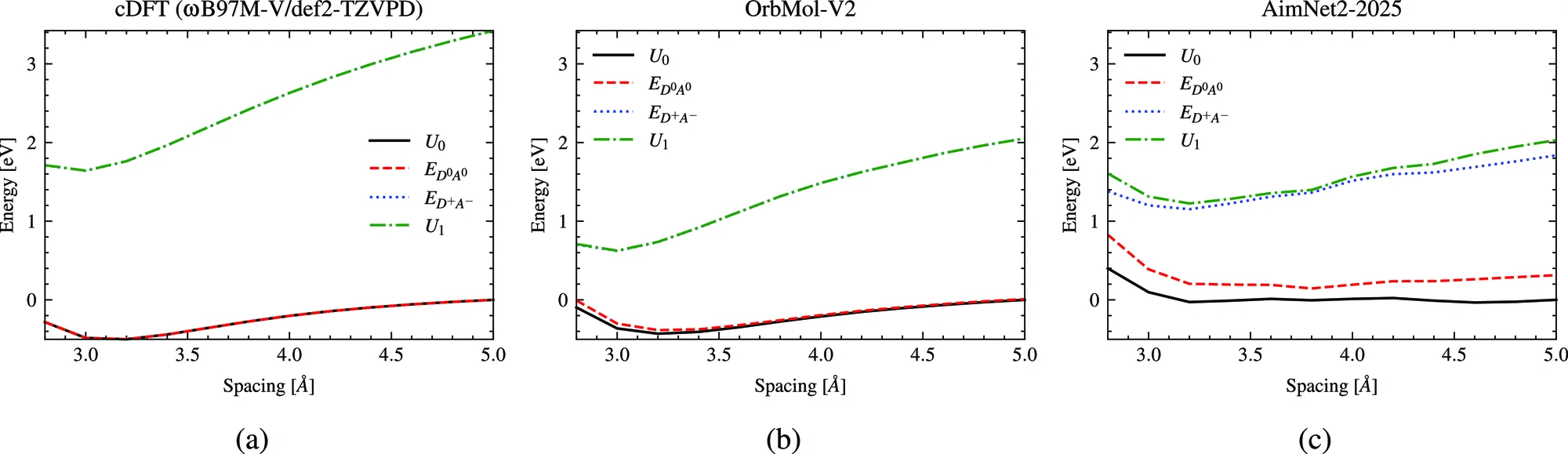
Adiabatic, (*U*_0_ and *U*_1_) and diabatic energies ( $E_{D^{0}A^{0}}$ , and $E_{D^{+}A^{-}}$ ) for the CT process in the A-TCNE complex using (a) cDFT, (b) AimNet2, and (c) OrbMol-V2. The energies are reported relative to the ground-state energy at 5 Å.

**Figure 5:**
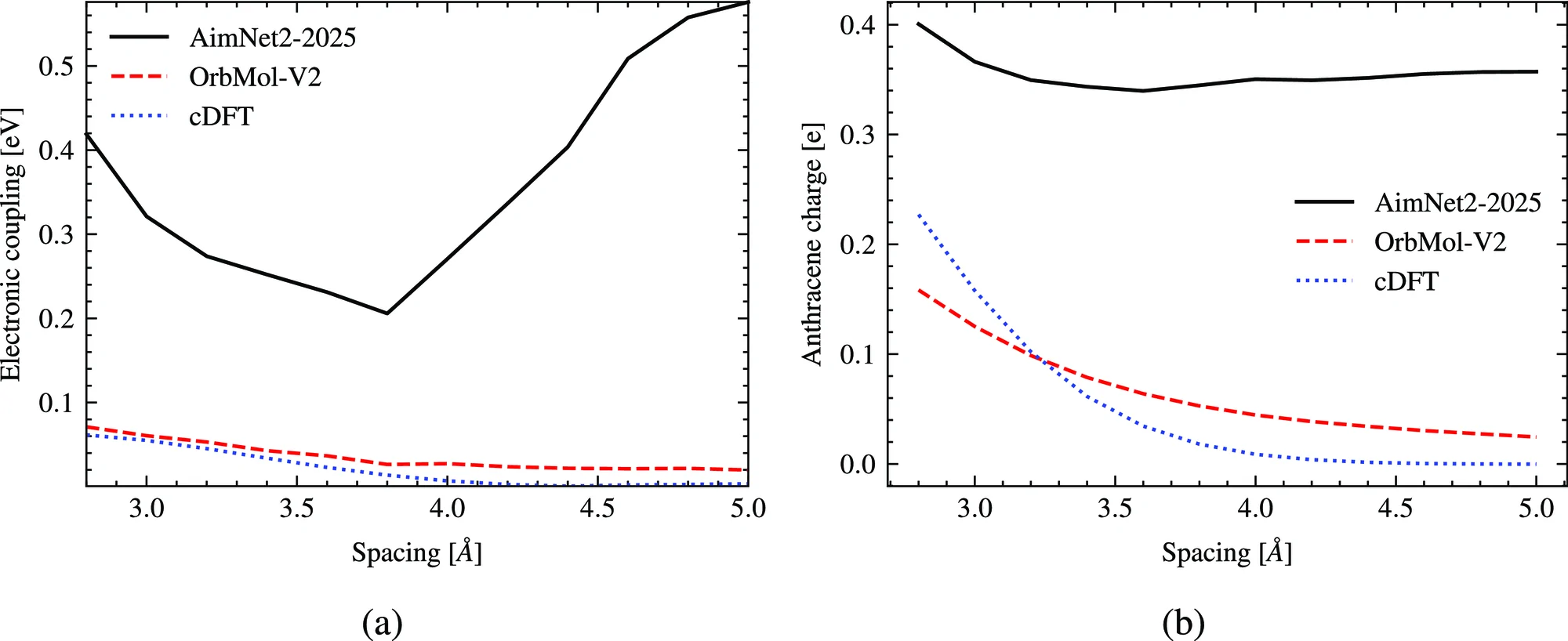
(a) The estimated *V*_12_ between the groups and (b) the net charge per fragment in the ground-state. Of note, the AimNet2 model fails to converge toward two neutral fragments while the DFT calculation trends toward each group having a net-zero charge.

After extracting the couplings from the adiabatic and diabatic states using ([Disp-formula eq4]), the AimNet2 model fails to capture the expected inverse relationship between the intermolecular distance and *V*_12_ (see Figure 5a). However, OrbMol-V2 is able to nearly match the *V*_12_ in the system. From the adiabatic energies, OrbMol-V2 produces an overall smoother energy surface compared to AimNet2. It is suspected that OrbMol-V2’s architecture is enabling better stability and connection between the fragments compared to AimNet2. The partial charge from AimNet2 are determined from the final layer of NQE (1) and similarly from the OrbMol-V2 latent charge layer. Extracting the Löwdin partial charges from the ground state DFT calculation reveals that the AimNet2 model does not exhibit a decaying fragment charge and rather converges toward a nonzero charge distribution between the two molecules (see Figure 5b). In contrast, OrbMol-V2 underestimates the net charge of the anthracene complex, which is likely contributing to the underestimating of the charge separated state. It should be noted that both models are only applicable for fragment spacings below the cutoff distance for each model, (5 Å AimNet2 and 6 Å for OrbMol-V2). It is expected that the short-range potential is important to capture the interfragment interactions for the CT process. For long-range CT reactions, a model with a larger cutoff or a global scale interaction layer, such as the AllScAIP,^35^ may be required to capture the interaction effects at large separation distances.

### 3.3 Amorphous Polymers—PE

The final case of comparison is for intrachain hole transport in amorphous PE. As system size increases, calculating the Marcus parameters can quickly become costly. The fragmentation of selected PE chains is shown in Figure 6. For calculating λ and Δ*G*, the chains were isolated from the full system and all carriers except the selected pair had constrained coordinates. For both *DA*_*r*_ and *DA*_*p*_, the selected carrier was constrained to a + 1 net charge while the rest of the chain was constrained to a 0 net charge. The coupling was calculated by using adjacent carrier constraints. When performing the geometry optimizations for λ and Δ*G*, only the carriers involved in the transport were allowed to relax. The remaining atoms in the chain were constrained to the initial positions. An example of the fragmentation of the chain is shown in Figure 6. Since small fragments represent a small number of the total (but could skew the distribution), those shorter than 5 CH units are not included in the analysis. This follows the choice of fragment sizes utilized by.^33^

**Figure 6:**
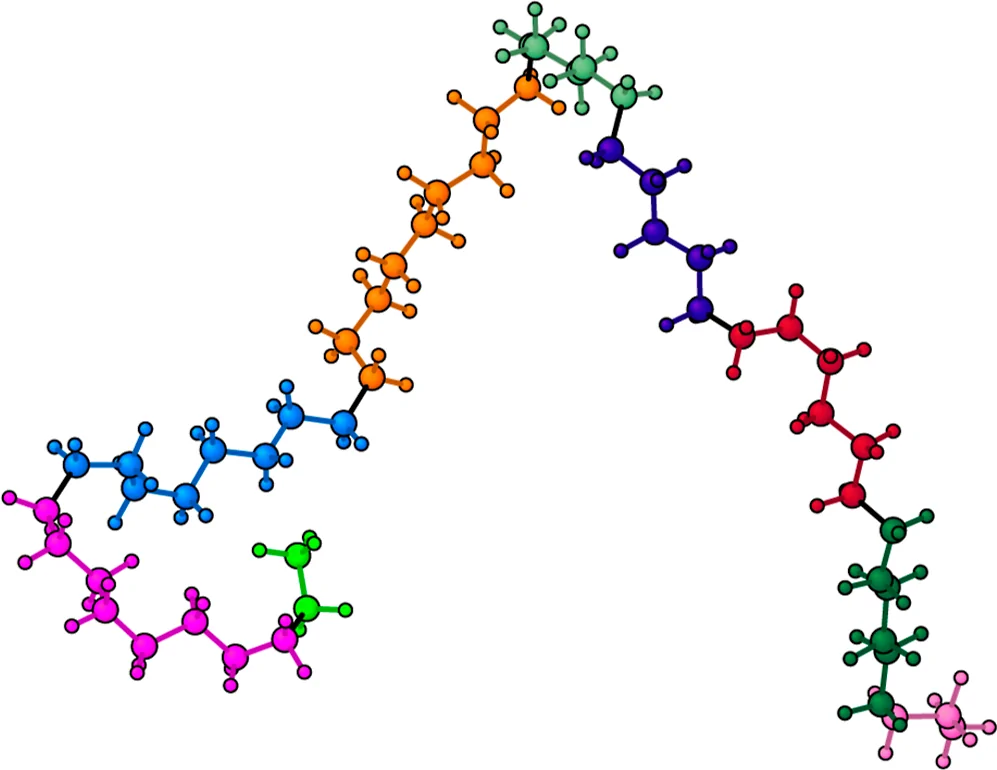
A selection of partitioned PE chains with the carrier segments highlighted. The segments are used as the states for the donor and acceptor when applying the constraints for the NcQeq simulation.

First, the electronic results are compared against reference cDFT for 10 of the 20 chains generated in Figure 7a which produced 79 coupling parameters. Only a portion of the total chains were used for this comparison due to the computational requirements for running cDFT for long PE chains. For both AimNet2 and OrbMol-V2, the models are linearly proportional to the target data. Furthermore, performing a linearly correction to the cDFT data, the models achieve a root-mean square error (RMSE) of 0.17 eV (see Figure 7b). It turns out that both models combined seem to capture reasonably the same underlying physics and are linearly proportional to one another (see Figure 7c). The linear corections were applied using Numpy 2.5.0 and SciPy 1.18.9.^36,37^ The Pearson (*r*) and Spearman (ρ) correlation metrics for the correleation are in Table 2. A scaling to the meV range may be required when using the coupling values in downstream modeling tasks to achieve the correct behavior since (4) is known to overestimate *V*_12_. Next, the overall distributions for the different Marcus parameters are shown in Figure 8. The mean values for λ of 0.9 and 1.13 eV for AimNet2 and OrbMol-V2, respectively, are within the ranges reported in.^33,38^ While the standard deviation of the Δ*G* is outside the ranges reported, the differences may be accounted for with several difference reasons. The difference in values is likely due to several confounding factors: (1) differences in modeling approaches, (2) lack of an explicit solvation model, and (3) level-of-theory differences. First, this work relies on the cDFT approach for determining Δ*G* and models the full polymer chain, while^33^ and^38^ use fragmentation approaches and the four-point method for determining Δ*G*. Second, the results presented here do not account for the effects of nearby chains or solvent molecules, whereas the other works use implicit solvation to approximate interactions with nearby chains. Finally, all approaches are based on different levels of theory, where^38^ uses B3LYP/Def2-SVP,^33^ uses B3LYP/DZVP, AimNet2 is trained on a combination of ωB97M-D3/def2-TZVPP and B97-3c with D3 dispersion corrections, and OrbMol-V2 is trained on ωB97M-V/def2-TZVPD. Further investigation is required to understand the functional and basis set effects on modeling the charge transport behavior is PE. Since, to the author’s knowledge, cDFT has not been applied to charge transport in polymeric materials, a deeper investigation into the behavior of the environment and settings correct geometry optimization and charge constraints is required.

**Figure 7:**
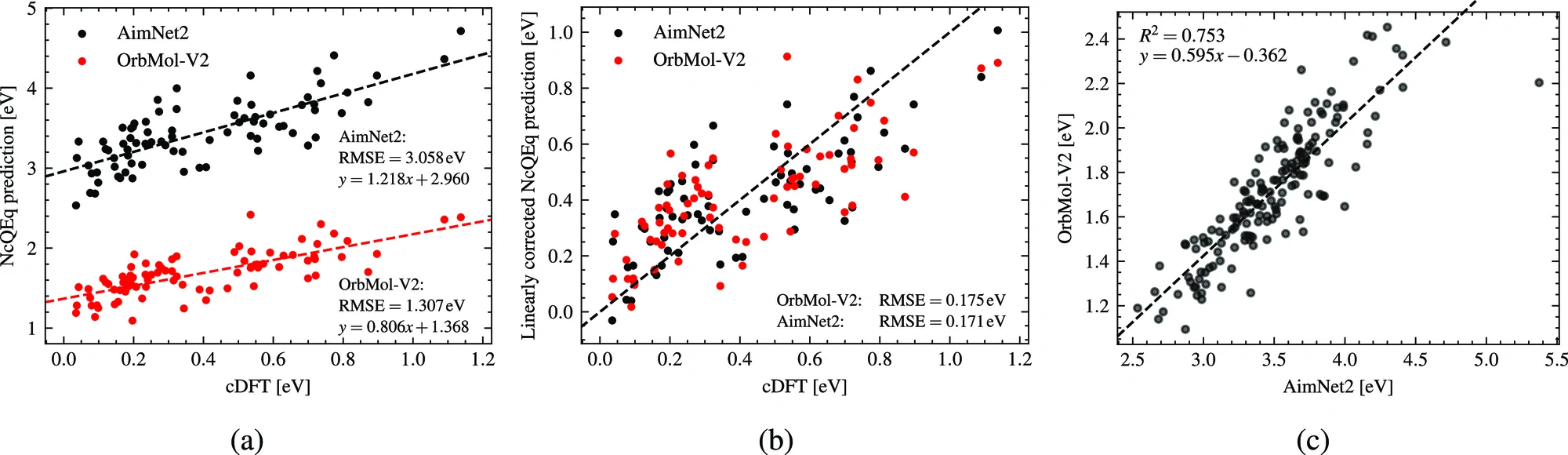
(a) When comparing the cDFT data to the results from both AimNet2 and OrbMol-V2 for 10 PE chains, both models produce results with an approximately linear relationship. (b) After applying a linear correction to the predictions, both models have root mean squared errors <0.2 eV. (c) Furthermore, both models are found to capture similar behavior are determined to be linearly related to one another. The linearly relationship eludes to the fact that both models have captured some concept of the physical charge redistribution required for the diabatic states.

**Table 2:** Correlation Metrics Produced for *V*_12_ Values Predicted by NcQEq and cDFT^a^

model	pearson *r*	spearman ρ
OrbMol-V2	0.748	0.746
AimNet2	0.766	0.739

a (*n* = 79 Matched carrier pairs per model).

**Figure 8:**
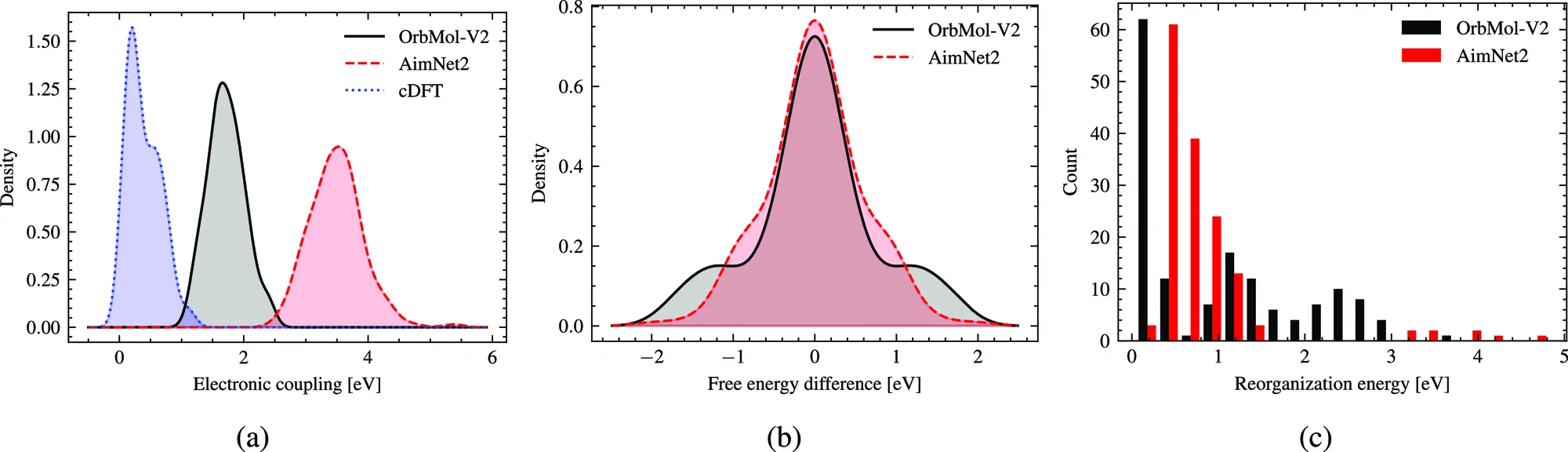
Distributions for the (a) *V*_12_, (b) Δ*G*, and (c) λ. These are based on the analysis of 20 PE chains (a) is presented unaltered (i.e., not adjusted based on the linear model). to distinguish the different predicted distributions. After filtering out small fragments <5CH_2_ units, the mean λ is 0.9 and 1.13 eV for AimNet2 and OrbMol-V2, respectively, which are within the range reported by.^33^ The standard deviation of the free energy differences is 0.6 and 0.75 for AimNet2 and OrbMol-V2, respectively. These values are outside the ranges reported in^33,38^ but may be due to constraint differences and environmental effects.

## 4 Conclusions

In this work, the concept of applying a constraint to NQE was introduced and found to be capable of estimating parameters related to CT. The approach assumes that a MLIP trained with partial charges is capable of learning the response of electrostatic changes. When compared to traditional DFT methods, this approach yields estimates for CT parameters significantly faster. The method relies on the short-range portion of the MLIP to adapt to the changes in the charge distribution in the material. Specifically, this approach is only applicable below the neighbourlist cutoff value on which the model was trained. To model long-range charge transport reactions, either models with larger cutoff values (>6 Å) or global interactions, such as those prosed in.^35^ Additionally, the NcQeq approach may not be suitable for systems outside the training distributions of the underlying model, requiring accurate modeling of orbital overlap, and/or requiring environmental or solvation effects.

From these results, the constrained approach appears to be applicable to inter- and intramolecular charge transport with systems ranging from charge separation in FA-AQ to intrachain hole transport in PE. In the case of A-TCNE, the AimNet2 model was found to be inaccurate at large separation distances and the ground state adiabatic charge separation was determined to be a contributing factor. The AimNet2 model failed to capture the convergence to neutral fragments with increasing distance and represents a potential mode for improvement in the model architectures or training data. In contrast, OrbMol-V2 was able to correctly predict the decaying *V*_12_ with distance. The larger cutoff value of the OrbMol-V2 model may be a contributing factor, which indicates that for NcQeq approaches, larger cutoff values may be preferable for accurate modeling.

Further, when studying intrachain coupling in PE, the NcQeq approach produces qualitative estimates for the intrachain hole–transport parameters. These results enable screening of desirable electronic parameters in bulk materials, such as amorphous polymers, which are typically time-consuming to model and predict, especially when considering multiscale factors.^39^ Based on the initial timings reported for AimNet2, the model’s runtime is comparable to force field methods. The *V*_12_ values predicted are applicable for initial screening and comparative analysis. With further validation and development (of both the NcQeq method and the charge predictions of the MLIPs), the introduced approach could be used for absolute predictions. Additionally, with the further development of models including more robust partial charge estimation schemes, such as those in MACE-Polar-1,^24^ it is expected that the additional constraints may be used to produce more accurate results.

NcQeq is presented as a proof-of-concept approach for predicting diabatic states from MLIPs trained only on adiabatic states. It may aid in estimating charge transport properties, enable comparative screening of new amorphous semiconducting polymers, and support the development of improved molecular complexes for photovoltaics. In addition, the approach provides insight into the learned behavior of the short-ranged potential of MLIPs. For CT, the generation of further data sets for comparison at high levels of theory may aid in the development of MLIPs capable of better predicting diabatic states. The future uses of a NcQeq approach will enable scaling charge transport simulations and developing novel materials and chemistries using the rapid estimations.

## Data Availability

The data and code will be made available upon a reasonable request to the authors.
